# Effect of Water to Cement Ratio on Properties of Calcium Sulfoaluminate Cement Mortars

**DOI:** 10.3390/ma17122806

**Published:** 2024-06-08

**Authors:** Małgorzata Gołaszewska, Jacek Gołaszewski, Bartosz Chmiela

**Affiliations:** 1 Faculty of Civil Engineering, Silesian University of Technology, Akademicka 5, 44-100 Gliwice, Poland; jacek.golaszewski@polsl.pl; 2 Faculty of Materials Engineering, Silesian University of Technology, Krasińskiego 8, 40-019 Katowice, Poland; bartosz.chmiela@polsl.pl

**Keywords:** calcium sulfoaluminate cements, w/c ratio, hydration heat

## Abstract

Calcium sulfoaluminate (CSA) cements are a promising alternative to Portland clinker, however, a thorough understanding of their properties is needed for their broader use in the industry. One of the topics that requires a good understanding is the effect of the w/c ratio on the properties of CSA cements. To this end, the aim of this paper was to provide research into the effects of a w/c ratio in the range of 0.45–0.6 on the properties of fresh and hardened CSA pastes and mortars. For fresh mortars, consistency and setting time, as well as plastic shrinkage tests, were conducted, and were complemented by hydration heat tests, carried out on pastes. For hardened mortars, tests of compressive and flexural strength and dry shrinkage, as well as SEM photography, were conducted. It was found that, regardless of a higher hydration rate, the increase in w/c ratio decreased flexural and compressive strength, as well as shrinkage, while increasing consistency, setting time, and hydration heat. Also observed was a significant decrease in strength between 3 and 7 days of curing in mortars with a high w/c ratio. It can be concluded that, regardless of the hydration rate, low w/c ratios in CSA mortars provide better properties than high w/c ratios.

## 1. Introduction

With the production of cement being responsible for 5–8% of global anthropogenic CO_2_ emissions, it is necessary to find ways to decrease its carbon footprint [1,2]. Due to the fact that the production of Portland clinker is the main source of CO_2_ in cement manufacturing, efforts focus mainly on finding ways to decrease the Portland clinker content in cement [3,4]. The easiest and most common way to decrease the carbon footprint of cement is by using additional cementitious materials, such as granulated blast furnace slag or fly ash, which are mostly by-products of industrial processes and, therefore, provide almost no CO_2_ emissions in cement production compared to clinker [3,5]. In addition to environmental benefits, the use of additional cementitious materials can positively affect numerous properties of cements and concrete, by enhancing durability, late strength, rheological properties, or decreasing shrinkage [6,7,8,9] The main problem with the use of additional cementitious materials in mixed cements is, however, their limited availability, especially with regard to the volume of cement production [10]. For this reason, there are other ways of reducing CO_2_ emissions from cement production, such as alternative binders [11]. One such material is CSA clinker, which is used to produce CSA cements.

Calcium sulfoaluminate (CSA) cements are considered to be a more ecological alternative to ordinary Portland cements (OPC) due to lower CO_2_ emissions during their production [12,13]. However, their popularization is held back by the fact that the production cost of CSA cements is currently higher than that of OPC, and, moreover, it is not as well described and researched [14]. In recent years, a lot of research has been conducted to explore the effects of using CSA cements on the properties of mortar and concrete, with the explicit aim of increasing the viability of its use in construction, however, many behaviors of CSA cements are not yet well documented. 

While both are hydraulic binders, CSA cement is significantly different to ordinary Portland cement. It is obtained by burning limestone, bauxite, and gypsum at a temperature significantly lower than that of OPC, which uses limestone and marls as input [15,16]. Due to the different inputs and the burning procedure, the phase composition also differs between the two types of cements. While OPC consists mostly of alite C_3_S, belite C_2_S, and tricalcium aluminate C_3_A, which hydrate into CSH phases [17], CSA cement composition is mostly based on ye’elimite (C_4_A_3_Ŝ), belite (C_2_S), and small amounts of calcium sulphate (CŜ), which hydrate into ettringite (C_6_AŜ_3_H_32_), stratlingite (C_2_AŜH_8_), monosulphate, and small amounts of CSH phase from the belite reaction [18,19]. 

Due to their differences in composition, CSA cement, regardless of its type, is characterized by a shorter setting time, lower shrinkage, and higher early strength than OPC [14,20,21,22,23,24,25]. 

Due to the fast hydration and high water demand of CSA cement, CSA pastes and mortars can exhibit rapid stiffening, and, thus, exhibit issues with workability. By increasing the w/c ratio, it is possible to decrease the viscosity and the yield stress of the mixture and, thus, improve the workability [26]. Moreover, one of the issues connected with the hydration process of CSA cement is the fact that the resulting ettringite requires much more water in the hydration process compared to the CSH phase. Stoichiometrically, the water to cement ratio (w/c) required for the Portland clinker to fully react is around 0.2 [27,28], while for the CSA clinker it was calculated to be over 0.6, and greater than 0.78 [29]. In OPC, any amount of water greater than 0.2 of cement mass is not strictly necessary, and an increase in the w/c ratio is a trade-off between better consistency and increased porosity, decreased compressive strength, and increased shrinkage [17]. However, it follows that, in the case of CSA cements, this relationship may not be unequivocal since the hydration of ettringite requires a large amount of water. It has been found that, while the reaction of ye’elimite is dependent on the calcium sulfate amount in the cement, hydration proceeds differently in an environment with high water content: when water is scarce, the products of the reaction are monosulphate (AFm) and aluminum hydroxide (1), while when water is readily available, ettringite (AFt) is the main output, together with monosulphate, hydrogarnet, and aluminum hydroxide (2), as can be seen below [30,31]:

C_4_A_3_Ŝ + 18 H → C_4_AŜ H_12_ + 2AH_3_, (1)

4 C_4_A_3_Ŝ + 80 H → C_6_AŜ _3_H_32_ + C_4_AŜH_12_ + 2C_3_AH_6_+ 8AH_3_, (2)

The effect of the w/c ratio on the properties of CSA cements was a topic of previous research. The possible effect of the water content on the setting of the CSA cements is not as unequivocal as it is in the case of the OPC cements. Increased w/c ratio has been found by Herrmann [25] to delay the initial setting time of CSA mortars, while tests by Doval et al. [30] have shown that ye’elimite hydration is faster for higher water/solids ratios. On the other hand, the effect of the w/c ratio on early hydration has been thoroughly tested, and findings are similar across different research. An increased reaction rate of mortars with CSA and high w/c ratio was shown by research by Wang and Song [32], Gołaszewska et al. [33], and Doval [30]. For Tang et al. [34,35,36], the research results indicated an increase in the heat rate of CSA cements with a higher w/c ratio and, consequently, an increase in the temperature of the samples with increased water content. 

However, the effect on compressive strength is not as clearly defined in the available sources. The negative effect of the high w/c ratio on the compressive strength of CSA mortars was observed by Dachtar [29] and Zhang et al. [37], whose research included samples with w/c ratios between 0.73 and 0.24. Additionally, porosity has been found to increase with the increase in the w/c ratio of CSA mortars, which provides possible grounds for the decrease in compressive strength of CSA mortars [34]. On the other hand, Wang and Song [32] have found that mortars with a w/c ratio below 0.4 can exhibit decreases in compressive strength if cured in water for more than 7 days, while samples with a w/c ratio of 0.48 did not exhibit similar behavior, providing evidence of a possible positive effect on compressive strength. 

In the case of the effect of w/c ratio on shrinkage, an increase in the w/c ratio in the range of 0.32–0.48 was linked to an increase in the chemical shrinkage of mortars with CSA cement [32]. It should be noted, however, that the information about the effect of the w/c ratio on different forms of shrinkage is very limited. 

There is also very little research available that presents a comprehensive look at the topic of the properties of CSA cements in relation to w/c ratio, rather than focusing on the testing of singular properties. Wang and Song [32] have tested CSA mortars with different w/c ratios in the range of 0.32 to 0.48. It was found that a higher w/c ratio increased the reaction rate; however, the higher w/c ratio caused a decrease in compressive strength, increased porosity, and shrinkage. It should also be noted that the tests were conducted on a w/c ration < 0.5, and, therefore, did not taking into consideration amounts of water close to the amounts which were calculated to be necessary for the full hydration of ye’elimite. Burris and Kurtis [38] conducted a study of the effect of the w/c ratio on the hydration and strength development of the belite calcium sulfoaluminate (CSAB) cement, which is characterized by a higher amount of belite (40–50%) than calcium sulfoaluminate cements (5–20%). Similarly, Koga et al. [39] and Pérez-Bravo [40] conducted research on the w/c ratio’s effect on Belite–Ye’elimite–Ferrite (BYF) cement. It was found that the lower w/c ratios (0.4) resulted in the hydration process stopping after the first few days, while for pastes with a higher amount of water, no such effect was observed. Additionally, strätlingite was observed in the pastes with a higher w/c ratio after 7 days of hydration, while for pastes with a w/c ratio of 0.4, the amount of strätlingite was significantly lower. A reduction in the w/c ratio also resulted in higher strength. Due to the differences between the composition of CSA cements and the belite-rich cements, based on the ye’elimite results of the tests, refs. [38,39,40] may not be fully applicable, however, they may show the possible direction of the effects of the w/c ratio on the properties of CSA cements. It was found that with the mixes with CSAB cement and w/c ratio in the range of 0.3–0.6, with the increase in w/c ratio the hydration was slightly delayed, cumulative hydration heat increased, and compressive strength decreased, however, a higher hydration rate can be achieved. 

The aim of the following research was, therefore, to provide a comprehensive and in-depth look at the effect of w/c ratios in the range of 0.45 to 0.6 on several properties of CSA mortars, including fresh mortar properties and hardened mortar properties, for a more complete understanding of the possible effects. For fresh mortar, consistency, setting time, and plastic shrinkage tests were conducted, as well as hydration heat tests being conducted on cement pastes, while, for hardened mortar, tests of flexural strength, compressive strength, and drying shrinkage were conducted, as well as a scanning electron microscopy SEM being used to observe changes in the microstructure over time. 

## 2. Materials and Methods

The research was conducted on mortars prepared according to EN 196-1 [41], however, instead of water–-cement ratio of 0.5, w/c ratio was in range of 0.45 to 0.6. In case of tests of hydration heat, additionally, w/c ratio of 0.40 was utilized. As a point of reference, mortar with ordinary Portland cement CEM I 42.5R (OPC) was used, with w/c ratio of 0.5.

Composition of commercially available CSA and OPC which were used in the research is presented in Table 1, with their basic properties listed in Table 2.

Standard sand was used, according to EN 196-1 [41], meaning that the sand had strictly repeatable grain size distribution of up to 2.00 mm and, therefore, grain size distribution variances for aggregate could be omitted in the discussion of results. 

To research the effects of w/c ratio on the properties of CSA mortars, tests were conducted on both fresh pastes and fresh mortars, as well as hardened mortars. Tests consisted of initial setting time, heat of hydration, early shrinkage, drying shrinkage, flexural and compressive strength, and scanning electron microscope (SEM) photography of microstructure. 

Mortar compositions used in the research are shown in Table 3. 

Initial setting time was tested on mortars according to a modified procedure from standard EN 196-3:2016 [42]. Automated Vicat apparatus was used, equipped with additional weight for mortar testing. The modification of procedure concerned the mortar composition, as due to w/c ratio being the main factor in the research, amount of water in the tested mortar was not adjusted to obtain standard consistency. The results were calculated as a mean value from 3 measurements. 

Hydration heat was tested on pastes, in an isothermal calorimeter TAM Air, (TA Instruments, New Castle, DE, USA) according to the standard PN-EN 196-11 [43]. Internal mixing procedure was used, which allowed us to measure the hydration heat from the moment of adding water to cement. Samples of 5 g of cement with corresponding amount of distilled water were prepared. The amounts of water were based on the w/c ratio and admixture content in the mortars, as seen in Table 1. Due to the lack of negative impact of low consistency of the paste on heat of hydration tests, w/c = 0.4 was also used for testing. Reference samples for isothermal testing were also prepared, using quartz sand in amount calculated to have the same heat capacity as each cement paste sample. Measurement lasted 72 h (3 days) from the moment of adding water to cement, and temperature was maintained at 20 °C. The hydration heat was calculated as a mean value of three calorimetric measurements. The differences between measurements for the same type of paste were no higher than 5%. 

**Figure 1:**
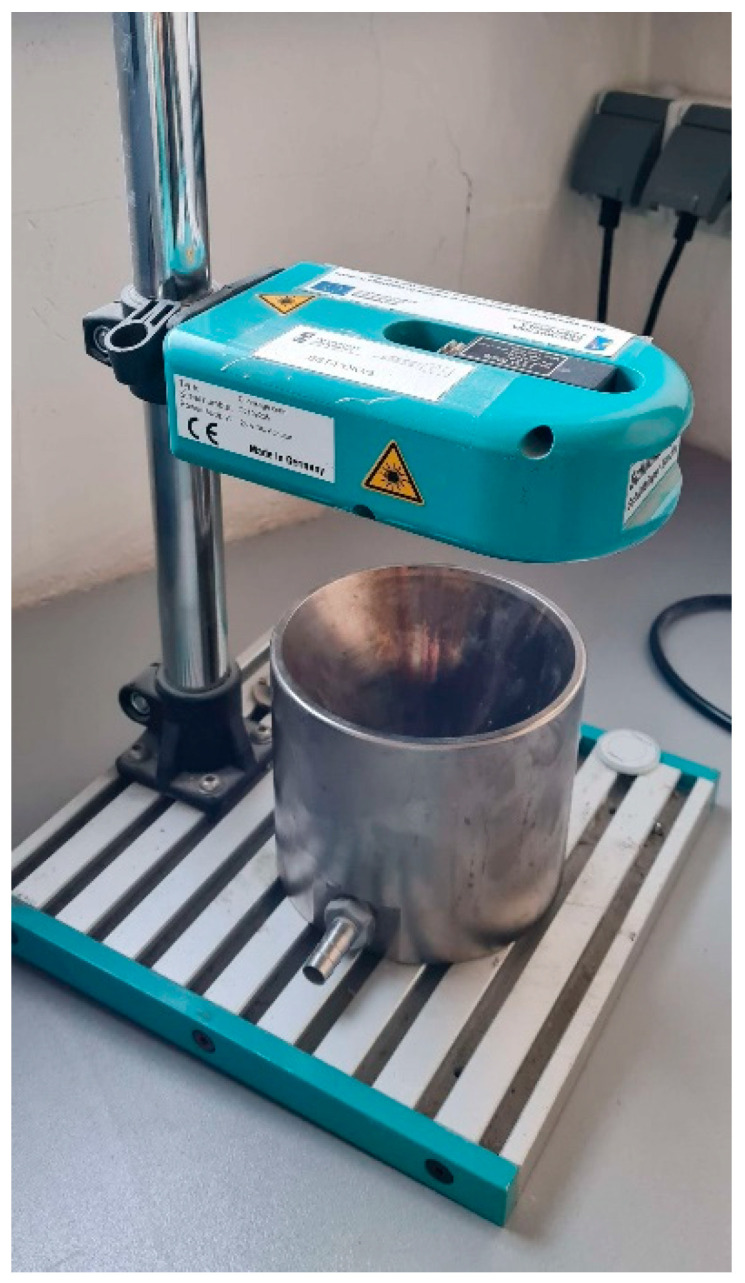
Early shrinkage testing setup—shrinkage cone.

Early shrinkage of mortars in the first 24 h from the moment of adding water to the cement was measured using shrinkage cone. The method, described in detail in [44,45], consists of a laser measurement system of a cone-shaped sample measuring the changes in the height of the cone (Figure 1). Mortar samples were mixed according to the procedure from standard EN 196-1:2016 [41], and then immediately placed into cone-shaped container of the shrinkage cone. The shrinkage cone was situated in the climatic chamber, at constant temperature of 20 °C and relative humidity of 60% during the whole measurement. Whole setup is shown in Figure 1. The measurement started 5 min from the mixing of water with cement and lasted 24 h. Results of early shrinkage testing were a mean value of three measurements. 

Drying shrinkage was tested using Graf Kaufman apparatus, according to standard EN 12617-4 [46]. Mortars were mixed according to the procedure found in standard EN 196-1:2016 [41], then poured into the 40 × 40 × 160 mm molds with aluminum caps inserted into the mortar. The samples were kept at a temperature of 20 °C and covered with foil to keep constant humidity for the first 24 h, then demolded and their length measured in Graf Kaufman apparatus, as an offset. After that, the samples were kept in climatic chamber keeping constant temperature 20 °C and 60% relative humidity. The measurement was repeated after 2, 3, 7, and 28 days, and relative change in length of the sample was calculated. For each mortar, three samples for shrinkage testing were prepared, and mean value was calculated to obtain the result of the test. 

Flexural and compressive strength of both CSA and OPC mortars was measured based on standard EN 196-1:2016 [41]. The mortars were mixed according to standard procedure and formed into prismatic samples measuring 40 × 40 × 160 mm. For first 24 h the samples were kept at 20 °C and covered with foil, then demolded and kept in water at a temperature of 20 °C until the date of their testing. Tests were performed after 1, 2, 3, 7, and 28 days after mixing the samples. For each test of flexural strength, 3 samples were used, and the 6 resulting samples were then used for compressive strength testing. Mean value of the tests was then calculated. To better gauge the relationships in the results, two-way ANOVA analysis was conducted for both flexural and compressive strength of CSA mortars. F-tests and *p*-values were calculated, where F-test calculates the ratio between group variances, and *p*-value is a measure of probability of obtaining the result if no effect were to be observed, meaning that the lower the *p*-value, the more probable it is that the observed effect is statistically significant. In the research, a commonly used *p*-value of 0.05 or less was considered to indicate the statistical significance of the effect. 

For CSA mortars with three different w/c ratios, 0.45, 0.5, and 0.6, as well as reference sample in form of OPC mortar, the SEM analysis of microstructure was conducted using S-3400N SEM (Hitachi, Tokyo, Japan) and Quanta FEI 250 FEG-SEM (ThermoFischer Scientific, Waltham, MA, USA) equipment. The applied acceleration voltage was 15 kV. Chemical composition was measured using electron probe microanalysis (EPMA) using energy-dispersive X-ray spectrometer (EDS) Thermo Noran (ThermoFischer Scientific, Waltham, MA, USA) and wavelength-dispersive X-ray spectrometer (WDS) Thermo MagnaRay (ThermoFischer Scientific, Waltham, MA, USA) operating at 15 keV of primary beam energy. CSA mortar samples measuring 40 × 40 × 160 mm were prepared according to standard EN 196-1:2016 [41], and then broken to obtain 40 × 40 × 20 mm sample, which was air-dried for 24 h before the measurement. To better show the phase formations in the microstructure, the sample was not polished, and the break plane was chosen for the analysis. SEM analysis was conducted after 1, 2, 3, 7, and 28 days from preparing the samples.

## 3. Results and Discussion

### 3.1. Setting Time

**Figure 2:**
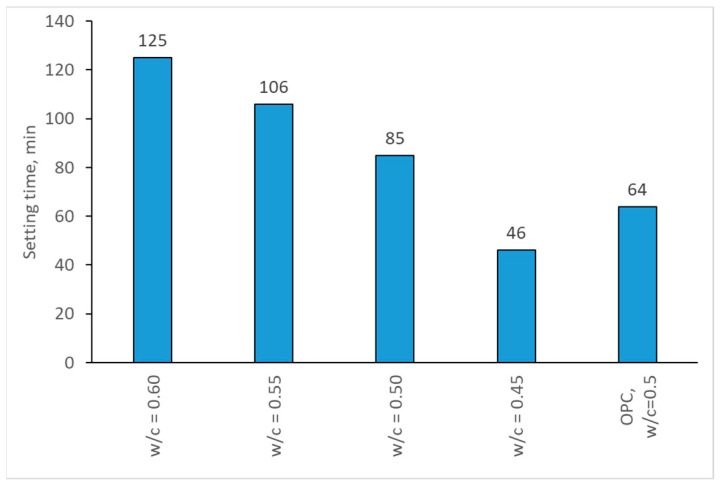
Setting time of CSA mortars.

The test results of the initial setting time of the CSA cements and OPC cement are presented in Figure 2. It can be observed that, with the increased water/cement ratio, setting time also increases. With the increase from 0.45 to 0.6, the initial setting time has almost tripled, amounting to around 80 min of the initial setting delay. This effect can be attributed to the physical effects connected with a high amount of water in the paste: cement particles are more spread out, and, thus, it takes longer for the hydration products to form a skeleton of a structure (which marks the initial setting time) as there is more space between the particles [47]. The increase in setting time cause by the increased w/c ratio in CSA mortars has also been observed by Wang and Song [32]. It should be noted that while the setting time for CSA cements is shorter than that for the OPC sample in the case of w/c 0.45, it is decidedly longer for other w/c ratios. While one of the benefits of the use of CSA cement is its fast setting time, the higher w/c ratio may be detrimental due to this effect. It should also be noted that the Vicat tests are mostly rheological in nature, and, therefore, the effect of a high water content may be misleading. 

### 3.2. Hydration Heat

**Figure 3:**
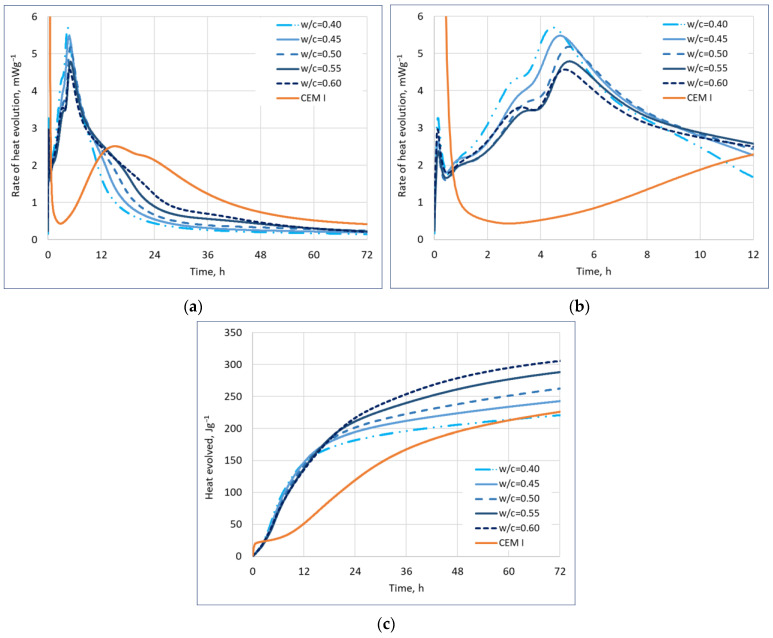
Hydration heat of CSA pastes with different w/c ratio: (**a**) rate of heat evolution in first 72 h, (**b**) rate of heat evolution in first 10 h, and (**c**) heat evolved.

The results of hydration heat tests are shown in Figure 3. 

As can be seen in Figure 3a,b, the early stages of hydration heat development and, thus, hydration are very similar in all samples with different w/c ratios since the end of the induction phase between the w/c ratios of 0.4 and 0.6 of the pastes differs by only 15 min. This effect is not consistent with the results of the initial setting time of CSA cements presented in Figure 2, however, it must be emphasized that the setting time measurement is mostly connected to the rheological properties of the mortar and its stiffening over time, and not necessarily directly to its hydration [48]. It can be, therefore, ascertained that the delay in initial setting time observed with the increase in w/c ratio is mostly a physical phenomenon, not chemical. Additionally, it can be noticed that the OPC hydration follows a different path than the CSA cement hydration—in this case, setting (taken as end of the induction phase) can be placed at 3 h for the OPC cement and ~10 min for the CSA cements (Figure 3b).

The difference between the hydration of the pastes with different w/c ratio can, however, be observed in the first hydration peak, which occurs around the 3 h mark from the start of the hydration. Its emergence can be linked to the initial dissolution of sulphates and the start of its reaction [49]. While the peak occurs for pastes with different w/c ratios at a similar time, with the difference between the earliest occurrence of the first main peak (for CSA w/c = 0.4) and the latest (for CSA w/c = 0.55) being 25 min. This means, taking into consideration the limitation of the testing equipment and sample size, that while the difference is present, it is not very significant. The intensity of the first main peak is also connected to the w/c ratio. The lower the w/c ratio, the higher the first main peak, however, it should be noted that for a w/c ratio between 0.5 and 0.6, the difference was not significant. This effect may be explained by the high level of dilution of cement particles in paste when more water is present, and, thus, a slower early hydration rate [50]. 

The main hydration peak was similarly delayed in the case of higher w/c ratios. The difference of 40 min was observed between the occurrence of the second main peak of hydration (for paste with 0.4 w/c ratio), and the latest (for paste with 0.55 w/c ratio), indicating the more pronounced effect of the high w/c ratio on hardening than on setting. 

The main difference in the development of hydration heat can be observed between the 10th and 30th hour of hydration. After the main peak, the hydration rate for the mortars with a w/c ratio of 0.4 drops quickly, with the reaction tapering out after ~24 h from the start of the reaction. However, as the water content in the paste increases, so does the heat emitted during that time, visibly showing emergence of another hydration peak. This effect may be linked to the second ettringite formation process. In the case of low w/c ratio, there is no water available for the reaction to occur, as ettringite (AFt) formation requires high amounts of water. In the case of higher w/c ratio, there is the possibility of water still being available for the reaction to start [51,52]. 

After the first 24 h, with the higher w/c ratio, the heat evolved is also increased (Figure 3c). The lowest heat evolved after 24 h for samples was obtained for the CSA pastes with a w/c ratio of 0.4, and the highest for a w/c ratio of 0.6, with a difference of 16%, which rose up to a difference of 28% after 72 h of hydration. This can be attributed to the additional peak related to the second ettringite formation process contributing to the cumulative hydration heat [51,52]. 

It can, therefore, be ascertained that the w/c ratio has a significant effect on the hydration heat and its development in CSA cement—to a lesser extend during early hydration, but to a high extend during the hardening phase. This effect is consistent with the previous tests of the CSA cement’s hydration [32,33]. 

### 3.3. Consistency

**Figure 4:**
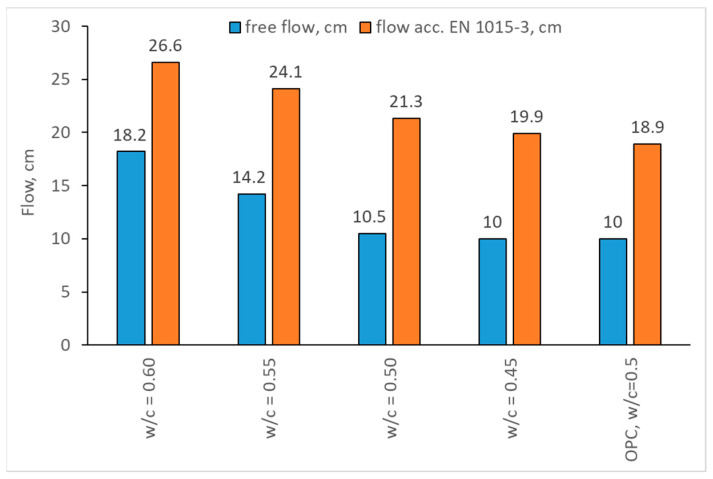
Consistency of CSA mortars with changing w/c ratio.

The results of consistency measurements are presented in Figure 4. 

The flow results are as could be expected—with a decrease in the w/c ratio, the flow and free flow are reduced, and for the w/c = 0.45 there was no free flow. It should be noted that the decrease in consistency with a decreasing amount of water was linear—for free flow, the decrease amounted to ~24% (22% decrease in flow between mortars with w/c = 0.6 and w/c = 0.55, and 26% decrease in flow between mortars with w/c = 0.55 and w/c = 0.50), while for the flow measured according to EN 1015-3 [53] the decrease in flow was in the range of 7–12%, with a decrease in w/c ratio of 0.05. CSA cements also exhibit a better consistency than OPC cements, having a higher flow with the same w/c ratio. 

### 3.4. Flexural and Compressive Strength of CSA Mortars

**Figure 5:**
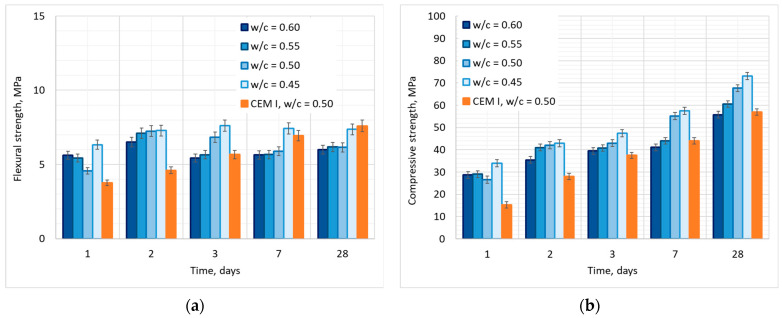
Effect of w/c ratio on (**a**) flexural and (**b**) compressive strength of CSA mortars with 95% confidence interval marked.

The results of the flexural and compressive strength tests and the statistical analysis ANOVA of the results in Figure 5 and Table 4. 

**Figure 6:**
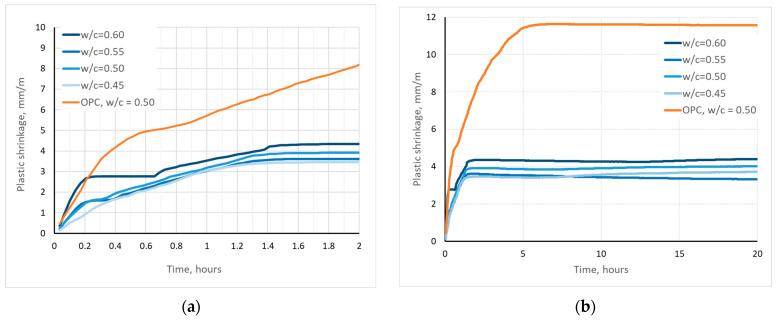
Effect of w/c ratio on plastic shrinkage of CSA mortars, (**a**) during first 2 h and (**b**) during first 20 h.

For all the tested CSA mortars, approximately 90% of flexural strength (85–97%) was obtained during the first 24 h of maturing. It can be observed that flexural strength after 28 days can be higher for mortar with a low w/c ratio, with the difference between flexural strength of CSA mortar with w/c = 0.45 and mortar with w/c = 0.6 amounting to 17%, while the difference in flexural strength of mortars with a w/c ratio of 0.6–0.5 is lower than 3%, and, therefore, is insignificant. This indicates that for a high amount of water in the mixture, the w/c ratio may not have a significant effect on flexural strength, and only for lower amounts of water (w/c = 0.45 and possibly lower) does the flexural strength increase. This can also be noticed in ANOVA analysis (Figure 6), where, while the w/c ratio is proven to be a significant factor, it can be noticed (Figure 6a) that the 0.95 confidence intervals indicate the close similarity of flexural strength of mortars with w/c = 0.6–0.5, but not w/c = 0.45. 

It should be noted that the development of flexural strength for all of the CSA pastes does not follow the same general path as for the OPC mortars (Figure 5a), as for all of the tested mortars, the highest compressive strength was obtained after 2–3 days of maturing, not 28 days. After that, a significant decrease in flexural strength was observed (up to 20%), followed by the slow process of further strength development. 

The w/c ratio also has a significant influence on compressive strength, which is supported by the *p* value of 0.000 at each test date (Table 4). Reducing the w/c ratio in CSA mortars results in an increase in compressive strength, even if this effect is less pronounced in the initial curing period, which can be noticed in Figure 5b and Figure 6b. This indicates that the high degree of hydration in CSA cements is not directly linked to the compressive strength increase, and other factors may be determinative. 

All CSA mortars are characterized by the high 1-day strength, which is significantly higher than in OPC mortars. Approximately 60% of the 28-day strength is obtained in the second day of maturing of the CSA mortars. After that, the strength development of the CSA mortars with low w/c ratios and OPC mortar is similar, while for the mortars with a high w/c ratio (w/c = 0.6 and w/c = 0.55), compressive strength development is inhibited on the 3rd and 7th day of maturing as the strength increase between 3rd and 7th day for mortars with a high w/c ratio is <10%, while for mortars with a low w/c ratio it is >20%. 

The effect of the decreased flexural and compressive strength in CSA mortars has been also observed by Brien et al. [54] and Burris and Kurtis [38]. 

The effect is difficult to explain, and several possible phenomena could be responsible for the inhibited strength development after the 3rd day. One of the possible explanations may be linked to the ettringite dehydration process described by Skoblinskaya and Krasilnikov [55,56]. When subjected to increased temperature, water is removed from the ettringite, transforming it into so-called metaettringite [57]. While the dehydration of ettringite has been a subject of numerous different research projects, there is no consensus as to what are the temperature and humidity conditions in which the process happens. While research by Pourchez et al. [58] and Zhou and Glasser [59] points to temperatures over 100 °C, the tests of Fridrichová et al. [60] show that even in laboratory conditions of 21 °C, the ettringite formed from hydration of ye’elimite can be unstable. Thus, it may be so that the decrease or stagnation in strength can be linked to this phenomenon as the temperature of the samples during hydration would be higher than 21 °C. Another possible explanation may be linked to the ettringite, which is expansive if a high amount of water is available, breaking the structure of the cement matrix [61]. It should also be noted that the possibility exists that the possible transformation of Aft into Afm could occur after the third day of hydration. Aft phases can be unstable in a high concentration, changing into Afm phases. The phase change from Aft to Afm has been found to significantly affect the compressive strength development of pastes, reducing or decreasing the rate of strength development [62,63]. This effect seems to be most probable at the time due to the changes in microstructure observed in conducted SEM testing presented further on. However, further testing is required to fully understand this effect. 

### 3.5. Shrinkage

The results of plastic shrinkage tests are shown in Figure 6. 

It can be seen that the early shrinkage of CSA mortars is much lower than that of mortars with CEM I. Moreover, the plastic shrinkage of CSA mortars stabilized after 1.4 h, while for the OPC mortar the stabilization happened only after 5 h. This can be explained by the different hydration process of OPC and CSA—the ye’elimite reaction is very fast, and, additionally, binds more water than the calcium silicate phases of OPC [18,19]. Therefore, not only does the setting of the CSA mortar happen earlier, but, also, less water is free for evaporation than in the OPC [17,64]. 

It must be noted, however, that the w/c ratio of CSA mortars has a strong effect on the plastic shrinkage, even if the difference is not as stark as it is between the shrinkage of OPC mortars and CSA mortars. For high w/c ratios, the shrinkage is higher. In case of w/c = 0.6, the plastic shrinkage during first 15 min is similar to that of OPC mortar. The effect of w/c ratio on plastic shrinkage can be attributed to the same mechanism as in the case of the Portland cement, that is, there is more free water available for evaporation [17]. 

**Figure 7:**
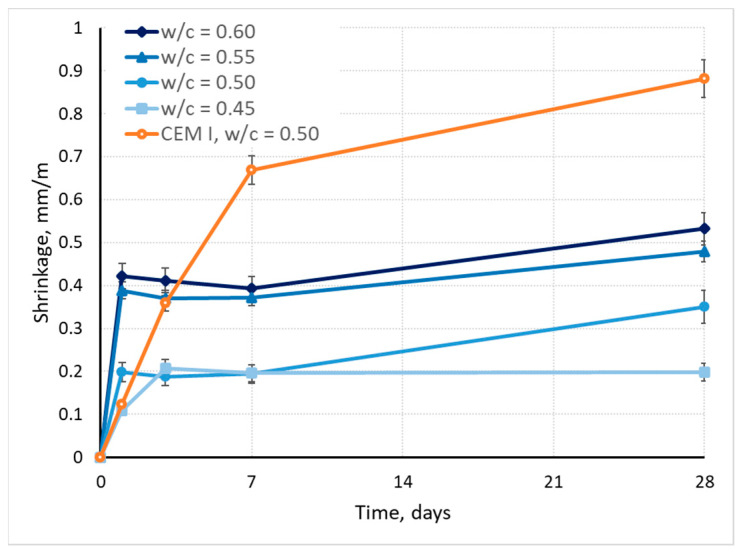
Effect of w/c ratio on drying shrinkage of CSA mortars.

The effect of the w/c ratio of CSA mortars on drying shrinkage is presented in Figure 7. 

The effect of w/c ratio of CSA mortars is more pronounced in the case of drying shrinkage than it was in the case of early shrinkage (Figure 7). The higher the w/c ratio, the higher the drying shrinkage. It should be noted, however, that the development of shrinkage is different in the case of CSA mortars than it is for OPC mortars. For CSA mortars, the drying shrinkage after 1 day of measurement amounts to >50% of 28 days shrinkage, while for OPC mortar, it was only 14%. For CSA mortars with a high water content (w/c = 0.6–0.5), after the first day, a small expansion occurs as the shrinkage decreases by 5%, and further shrinkage development occurs only after 7 days. In the case of CSA mortars with a low water content (w/c = 0.45), shrinkage is highest on the second day, and no development can be noticed after 7 days. This effect closely mirrors the effect of w/c ratio on flexural strength, indicating that the dehydration of ettringite during drying, and subsequent phase changes (ettringite into metaettringite) [55,56], may also affect the volume of the mix, lending credence to the theory about ettringite expansion in hardened mortar affecting the strength of mortars during the 3rd–7th day of hardening. This effect differs with differences in water content, as the phase composition is dependent on the water content in the matrix. 

It should also be noticed that in comparison to OPC mortar, CSA mortars are characterized by lower shrinkage, by 40% and more. 

### 3.6. Microstructure of CSA Mortars

**Figure 8:**
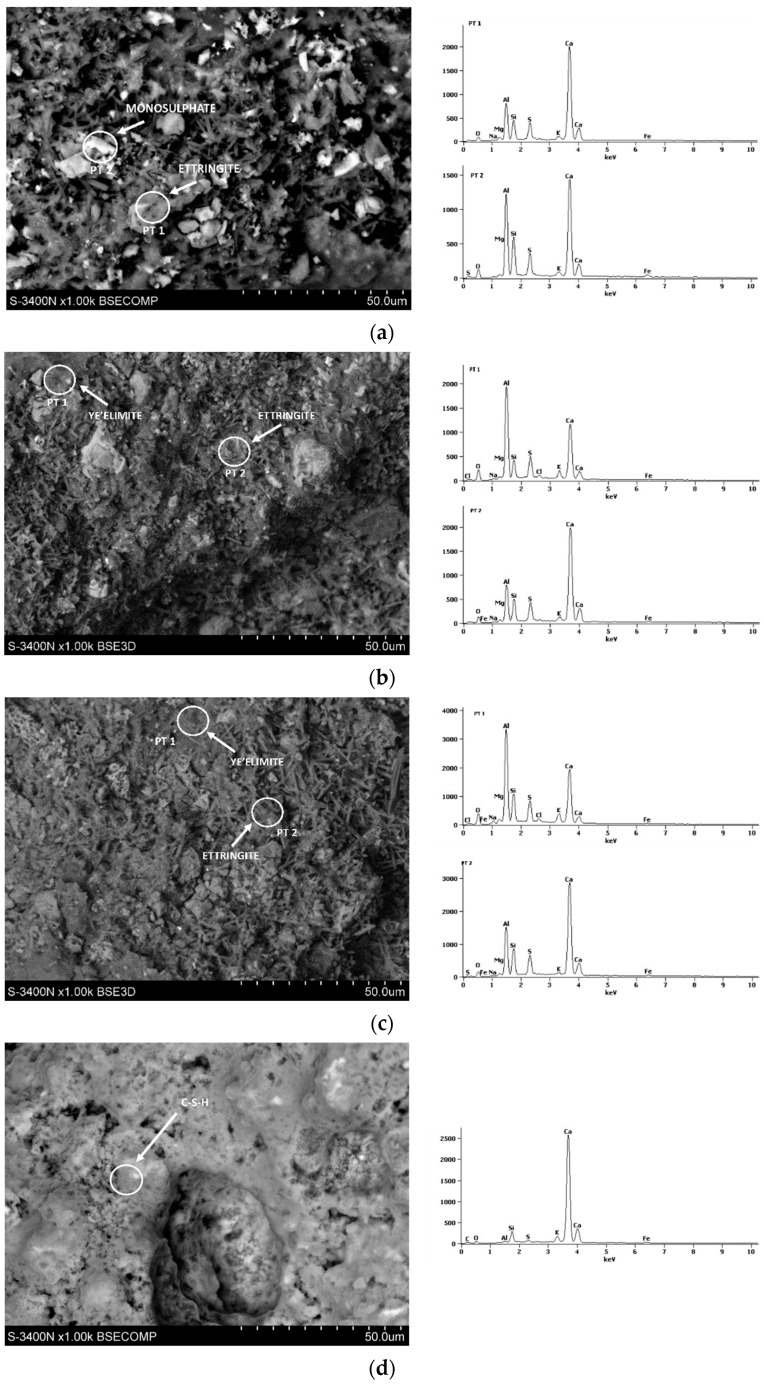
SEM photography with area SEM-EDS test results of mortars after 1 day: (**a**) CSA w/c = 0.45, (**b**) CSA w/c = 0.5, (**c**) CSA w/c = 0.6, and (**d**) OPC w/c = 0.5.

**Figure 9:**
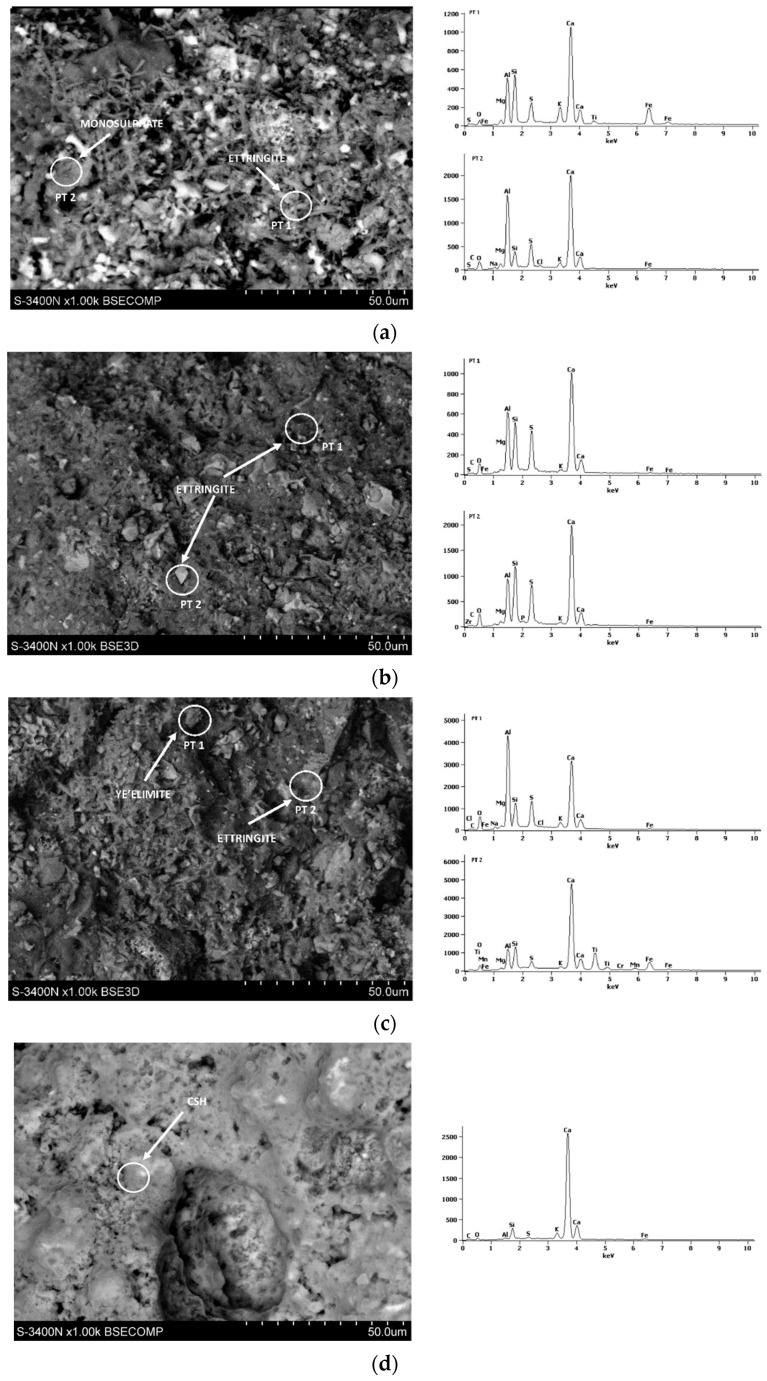
SEM photography with area SEM-EDS test results of mortars after 2 days: (**a**) CSA w/c = 0.45, (**b**) CSA w/c = 0.5, (**c**) CSA w/c = 0.6, and (**d**) OPC w/c = 0.5.

**Figure 10:**
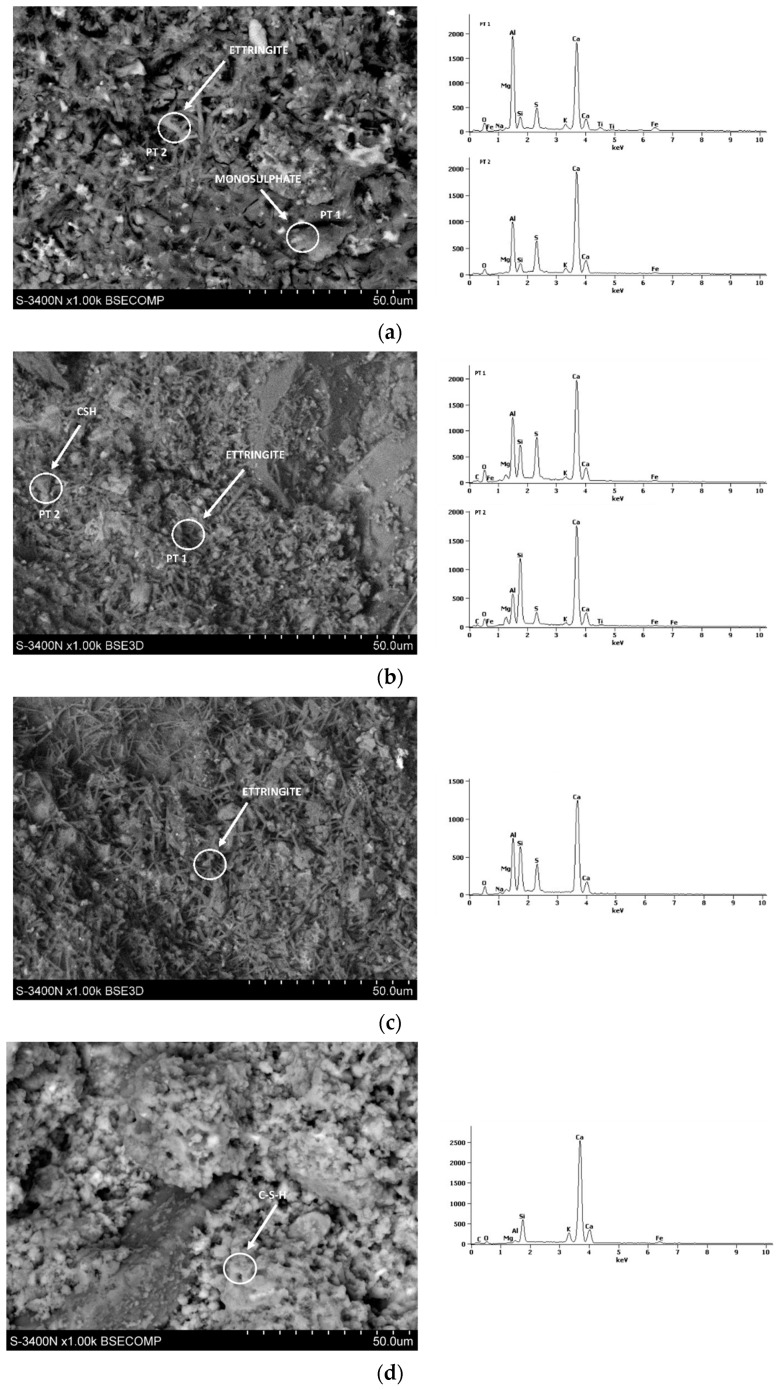
SEM photography with area SEM-EDS test results of mortars after 3 days: (**a**) CSA w/c = 0.45, (**b**) CSA w/c = 0.5, (**c**) CSA w/c = 0.6, and (**d**) OPC w/c = 0.5.

**Figure 11:**
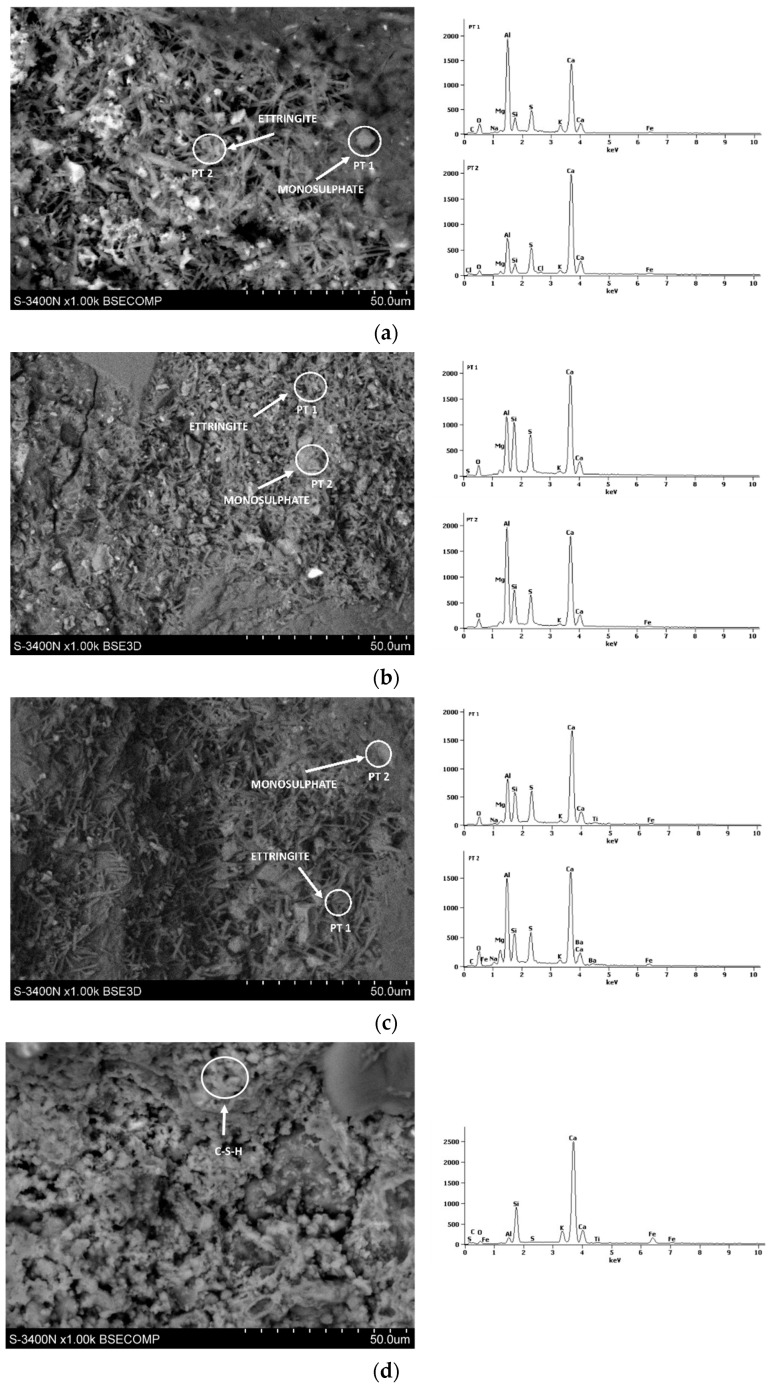
SEM photography with area SEM-EDS test results of mortars after 7 days: (**a**) CSA w/c = 0.45, (**b**) CSA w/c = 0.5, (**c**) CSA w/c = 0.6, and (**d**) OPC w/c = 0.5.

**Figure 12:**
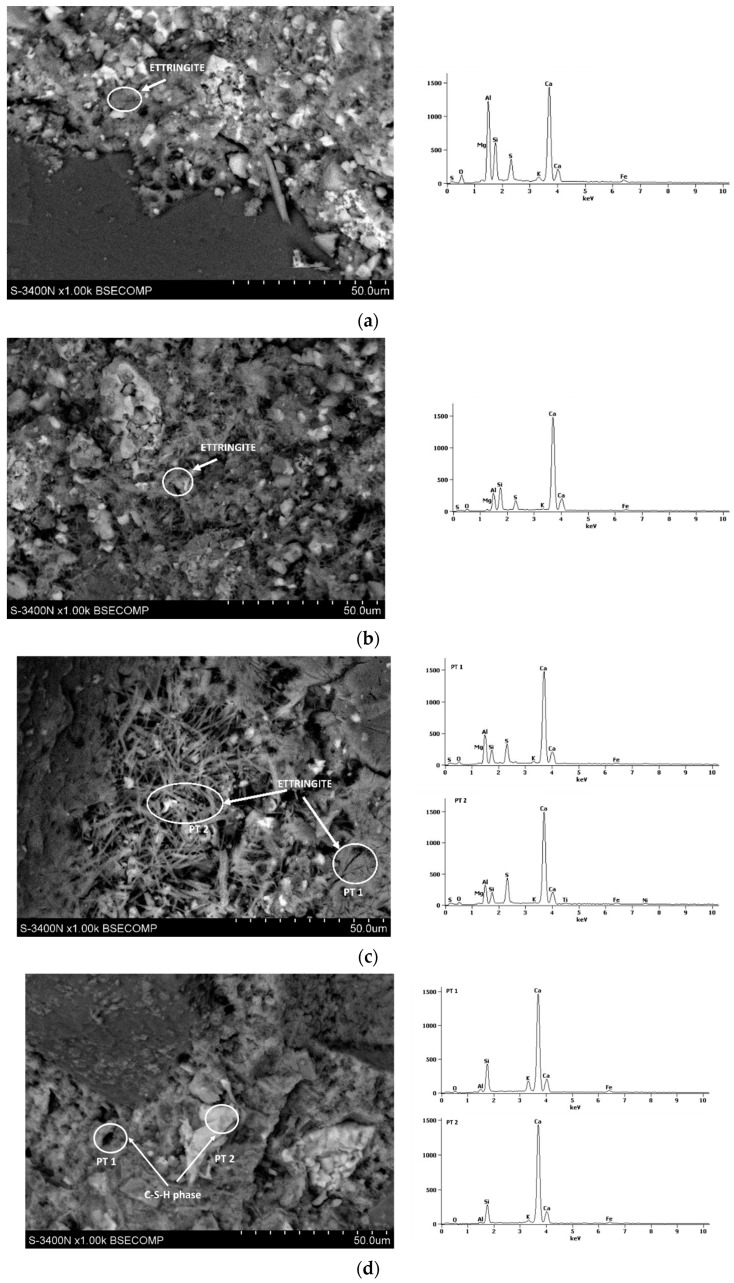
SEM photography with area SEM-EDS test results of mortars after 28 days: (**a**) CSA w/c = 0.45, (**b**) CSA w/c = 0.5, (**c**) CSA w/c = 0.6, and (**d**) OPC w/c = 0.5.

Figure 8, Figure 9, Figure 10, Figure 11 and Figure 12 show the SEM photographs of the mortar’s microstructure. The microstructure has been tested after 1, 2, 3, 7, and 28 days, and w/c ratios of 0.40, 0.50, and 0.60 were used for CSA cements, and a w/c = 0.5 for the reference sample of OPC. For each SEM photograph, chosen points were tested by energy-dispersive X-ray (EDS) analysis, providing spectrums which allowed us to identify phases present in the samples, and, thus, perform more in-depth analysis.

It can be clearly seen that the structure is significantly different between the OPC mortar sample and the CSA mortar samples. This is expected due to the fact that the exact hydration process is completely different in the case of CSA cements and OPC. In the case of CSA, the main phase that can be observed in all of the samples is ettringite. Ettringite can be observed mostly in the form of long needles, which can be easily seen in CSA samples, and which appear in significantly smaller amounts in the OPC sample. For the OPC samples, the main phase present is CSH, which was also expected. The CSH phase observed in the tested samples was in the form of dense, flock-like forms. The CSH phase also sporadically appears in the CSA samples, however, it is in significantly smaller amounts than in the OPC samples and was only observed in the samples after 3 days of hydration. It is consistent with the slower hydration rate of the CSH phase in comparison to the AFt and AFm phases. 

For early CSA samples (day 1), particles of unreacted ye’elimite can be noticed (Figure 9b,c). Ye’elimite traces were not found in the 28-day samples, showing that the hydration rate was quite high for each of the samples, regardless of whether it was close to the stochiometric amount of water present in the sample. 

The w/c ratio of the CSA pastes has a visible effect on the microstructure. It can be noticed that, in the first days of hydration (Figure 8, Figure 9 and Figure 10), the cement paste with the w/c ratio of 0.6 has a more dense ettringite formation, with a high amount of short needles, while the structures of the 0.50 and 0.40 exhibit ettringite forms which are longer, but less dense. It can be, however, noticed that the high amount of ettringite needles remains for samples with a w/c ratio of 0.6 after 7 and 28 days (Figure 11 and Figure 12), while the microstructure of samples with w/c ratios of 0.40 and 0.50 get denser. This is in line with the effect of the w/c ratio on compressive strength, where the high water content in the CSA mortars resulted in lower strength than in mortars with a low w/c—it can be expected that a loose structure will result in lower compressive strength. 

Additionally, monosulphate had been observed in the 0.4 samples since day one, but was not as easily found in the samples with higher w/c ratios. This is consistent with the chemical reaction of ye’elimite in differing amounts of water as, when the water availability is lower, more monosulphate is produced during the ye’elimite reaction [30,31]. 

Traces of monosulphate were found in samples with a w/c ratio of 0.5 and 0.6, tested at 7 days, which were not found in samples tested between 1 and 3 days. This may be consistent with the hydration heat results, providing a basis for the AFt transformation into AFm at later dates. This also points to, as discussed earlier, the possibility of AFm emergence being responsible for the decrease in the flexural and lack of development of compressive strength of the samples with higher w/c ratios. 

## 4. Conclusions

The presented research into the effect of w/c ratio on chosen properties of CSA mortars led to the following conclusions:

- Similarly to the OPC mortars, with the increase in w/c ratio, the initial setting time of CSA mortars increases due to the increased distance between cement particles.
- With the increase in the w/c ratio, the heat evolved also increases due to the higher rate of hydration. It must be noted, however, that a lower w/c ratio in CSA pastes leads to faster hydration and a higher main hydration peak; however, the induction phase is similar for all mortars, indicating that the difference in initial setting time observed during the Vicat test was based more on physical effects, rather than the hydration speed.
- An increase in water content in CSA mortar leads to a proportional increase in consistency.
- Both flexural and compressive strength are affected by w/c ratio, and, with the higher w/c ratio, a lower 28-day strength can be expected.
- A decrease in compressive and flexural strength was observed after 3 and 7 days, which may be linked to the phase change from the AFt to the AFm phase.
- CSA cements are characterized by a lower plastic and drying shrinkage than OPC cement due to the differences in their composition.
- The w/c ratio affects both plastic and drying shrinkage of CSA cements, and, with the increase in w/c ratio, the shrinkage also increases due to the higher water content.
- Differences were observed in the microstructure of CSA mortars with different w/c ratios, confirming that, after 3 and 7 days, the structures of CSA pastes with high amounts of water are less dense than those with a lower w/c ratio.

It can, therefore, be concluded, that, regardless of the hydration rate, low w/c ratios in CSA mortars provide better mechanical properties than high w/c ratios. Moreover, if a high amount of water is available in the matrix, possible negative effects can be observed between the 3rd and 7th day of hardening. This phenomenon may be linked to the phase change from the AFt to the AFm phase; it, however, requires further research.

## Figures and Tables

**Table 1:** Composition of CSA cement and OPC.

Cement	Constituent [%]									
	LOI	SiO_2_	Al_2_O_3_	Fe_2_O_3_	CaO	MgO	SO_3_	Na_2_O	K_2_O	Na_2_O_eq_
CSA	0.46	9.2	28.1	1.52	39.2	3.5	11.4	0.08	0.35	-
OPC	2.44	20.6	4.7	2.8	64.4	1.2	2.8	0.2	0.4	0.46

**Table 2:** Basic properties of CSA cement and OPC CEM I.

Cement Property	Unit	Value for	
		CSA	OPC
Initial setting time	min	45	185
Soundness of cement, by Le Chatelier’s method	mm	1	0.3
Compressive strength:			
at 2 days	MPa	42.0	28.06
at 28 days	MPa	67.7	57
Specific surface area	cm^2^/g	5500	4250

**Table 3:** Compositions of mortars used in the research.

Mortar Type	Cement [g]	Water [g]	Sand [g]
OPC w/c = 0.5	450	225	1350
CSA w/c = 0.60		270	
CSA w/c = 0.55		247.5	
CSA w/c = 0.50		225	
CSA w/c = 0.45		202.5	
CSA w/c = 0.40		180	

**Table 4:** Calculated F and *p* values from Anova analysis of influence of w/c ratio on flexural and compressive strength of CSA mortars.

Time	Flexural Strength		Compressive Strength	
	F	*p*	F	*p*
1 day	34.81	0.000	21.20	0.000
2 days	1.579	0.000	14.64	0.000
3 days	47.35	0.269	20.93	0.000
7 days	89.11	0.000	66.04	0.000
28 days	10.74	0.004	52.71	0.000

## Data Availability

The original contributions presented in the study are included in the article. Further inquiries can be directed to the corresponding author.
